# Inhibitory Circuits in the Basolateral Amygdala in Aversive Learning and Memory

**DOI:** 10.3389/fncir.2021.633235

**Published:** 2021-04-30

**Authors:** Madhusoothanan B. Perumal, Pankaj Sah

**Affiliations:** ^1^Queensland Brain Institute, The University of Queensland, Brisbane, QLD, Australia; ^2^Joint Center for Neuroscience and Neural Engineering, Southern University of Science and Technology, Shenzhen, China; ^3^Department of Biology, Southern University of Science and Technology, Shenzhen, China

**Keywords:** parvalbumin, somatostatin, chandelier, axo-axonic, fear, GABAergic, anxiety, stress

## Abstract

Neural circuits in the basolateral amygdala (BLA) play a pivotal role in the learning and memory formation, and processing of emotionally salient experiences, particularly aversive ones. A diverse population of GABAergic neurons present in the BLA orchestrate local circuits to mediate emotional memory functions. Targeted manipulation of GABAergic neuronal subtypes has shed light on cell-type specific functional roles in the fear learning and memory, revealing organizing principles for the operation of inhibitory circuit motifs in the BLA.

“*Unfortunately, nature seems unaware of our intellectual need for convenience and unity, and very often takes delight in complication and diversity.”*

Santiago Ramón Y Cajal, Nobel lecture 1901

## Introduction

Classical anatomical studies by Cajal and Golgi shed light on the morphological diversity of neurons in the brain. Cajal in particular was intrigued by the increase in number and complexity of “short axon neurons” up the evolutionary scale and concluded “.*functional superiority of the human brain is intimately bound up with the prodigious abundance and the unusual wealth of forms of the so called neurons with short axons*” (Yuste, 2005). These short axon neurons were subsequently found to release gamma-aminobutyric acid (GABA) at their axon terminals, the main inhibitory neurotransmitter in the mammalian brain, establishing these cells as inhibitory interneurons (Krnjevic, 1974). GABAergic neurons only comprise ~10–20% of the total population of neurons in the cortex, hippocampus and amygdala (McDonald, 1992; Freund and Buzsaki, 1996; Ascoli et al., 2008; Tremblay et al., 2016), but their activity tightly controls local network activity including the generation of large-scale network oscillations, such as gamma oscillations and sharp wave ripples, associated with learning and memory (Buzsaki, 2001; Roux and Buzsaki, 2015).

Information processing in the brain requires temporally organized activity in neural circuits (Harris, 2005). The identification of molecular markers to classify different types of interneurons, is revealing that the GABAergic neurons are an incredibly diverse population of cells, that have distinct physiological properties and form different types of local circuits (Ascoli et al., 2008; Tremblay et al., 2016). As a result, interneurons form specific circuit motifs to precisely control the timing of activity in mammalian circuits, and thus sculpt the temporal structure of network activity (Buzsaki, 2001; Woodruff and Sah, 2007a). Here we review the diversity of GABAergic neurons and their local circuits in the rodent basolateral amygdala (BLA), a mid-temporal lobe structure that plays a pivotal role in processing emotionally salient experiences, particularly aversive ones. The BLA is a cortical like structure, and similar to other cortical regions, local microcircuits are formed by excitatory glutamatergic neurons that make ~80% of total neuronal population and GABAergic interneurons (Sah et al., 2003; Tovote et al., 2015). These circuits generate a myriad of oscillatory network activities with distinctive frequency bands that play key roles in the acquisition, storage and consolidation of emotionally salient memories (Pare et al., 2002; Ponomarenko et al., 2003; Paz and Pare, 2013). In the BLA, many glutamatergic neurons resemble their cortical counterparts with a classical pyramidal soma, thicker apical dendrite and a spiny dendritic tree (McDonald, 1982). However, glutamatergic neurons in the lateral amygdala often lack pyramidal shaped soma or clear basal and apical dendrites as in the cortex. As glutamatergic neurons constitute the main neuronal subtype in the amygdala, they are referred as principal neurons (PNs) (Faber et al., 2001; Sah et al., 2003). In contrast, GABAergic neurons comprise of a diverse population of cells with different somatic morphologies such as triangular, oval, round or square shaped somata with bipolar or tuft dendrites that are either aspiny or sparsely spiny (McDonald, 1982). Similar to cortical regions (Kepecs and Fishell, 2014; Huang and Paul, 2019), GABAergic neurons in the BLA have been classified into a number of subtypes based on the expression of cytosolic markers, and their local connectivity (Spampanato et al., 2011; Capogna, 2014) and intrinsic electrophysiological properties (Sosulina et al., 2010). Apart from the presence of cytosolic markers, GABAergic neurons in the cortex and hippocampus, have also been separated based on the distribution of their local connections along organized layers (e.g., double bouquet cells in the cortex). With some principal neurons lacking a distinctive apical dendrite, and the fact that cyto-architectonically, the BLA lacks any recognizable laminar organization, comparison of morphological subtypes with their cortical or hippocampal counterparts is often not straightforward. However, oscillatory network activities in the BLA display similar frequency bands to those in the cortex and hippocampus suggesting functionally similar microcircuits may operate across these brain regions to mediate distinct cognitive functions (Pare et al., 2002). Here we review GABAergic neuronal subtypes in the BLA, their local circuit connectivity and their putative functional roles in aversive learning and memory.

## Diversity of GABAergic Neurons

**Figure 1:**
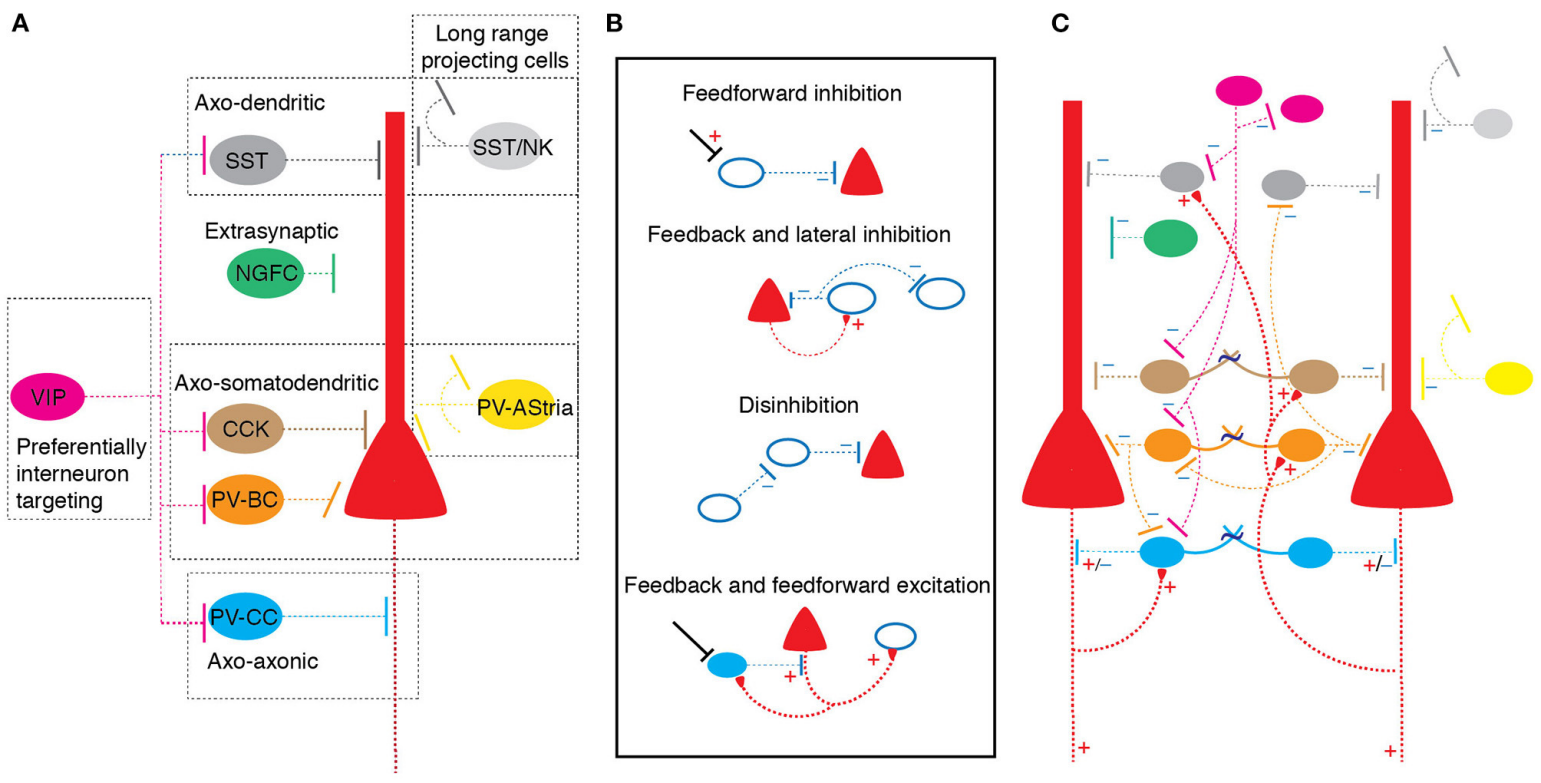
GABAergic neuronal diversity and circuit organization in the basolateral amygdala. **(A)** Types of GABAergic neurons color coded based on cytosolic protein expression: Somatostatin (SST), Neurokinin 1 (NK), Vaso intestinal peptide (VIP), cholecystokinin (CCK), Parvalbumin basket cell (PV-BC) and Parvalbumin chandelier cell (PV-CC) and Neurogliaform (NGFC). Dotted box indicates classification of groups based on synaptic target on principal neurons. Neurons with distinct cytosolic proteins tend to make distinct synaptic connections. **(B)** Schematic showing organization of circuit 'motif's formed on principal neurons and interneurons in the BLA. Bottom filled cell indicate excitatory axo-axonic chandelier interneuron; “+” excitatory connection, “–” inhibitory connection. **(C)** Schematic of a simplified BLA circuit model shows arrangement of different interneuron subtypes and putative circuit motifs shown in **(B)**.

Immunohistochemically, using expression of calcium binding proteins, GABAergic neurons in the BLA can be firstly be divided into two non-overlapping populations that express either calbindin or calretinin (McDonald and Mascagni, 2002). These neurons cells are then further classified based on the expression of other calcium binding proteins, neuropeptides or transmitter receptors into: (a) parvalbumin (PV), (b) somatostatin (SST), (c) cholecystokinin (CCK), (d) neuropeptide Y, (e) vaso-intestinal peptide (VIP), and (f) neurokinin 1subtypes (Kemppainen and Pitkanen, 2000; McDonald and Mascagni, 2002; Mascagni and McDonald, 2003; Sreepathi and Ferraguti, 2012) (Figure 1). To some extent, GABAergic neurons containing distinct cytosolic protein expression also target specific post-synaptic neuronal compartments of principal neurons. For example, PV+ and CCK+ neurons form basket-type synapses on the soma and/or proximal apical dendrites while SOM+ interneurons tend to target the distal dendritic tree of principal neurons (McDonald and Mascagni, 2001; Muller et al., 2006, 2007). However, GABAergic neurons with the same cytosolic marker proteins also display distinctive synaptic targeting. For example, while most PV+ interneurons make somatic basket synapses on principal neurons, a small proportion of PV+ neurons (<5%), named axo-axonic or chandelier neurons, form specialized “cartridge” type axo-axonic synapses at the axon-initial segment (McDonald and Betette, 2001). VIP+ terminals are less specific in their post-synaptic target selection, though this represents a heterogeneous group of CCK+ and CCK- terminals (Muller et al., 2003). In this schema, synaptic targeting distinguishes cells that innervate either the somatic compartment, the dendritic tree or the axon initial segment of principal neurons. In addition, some GABAergic neurons preferentially innervate other GABAergic neurons, some display non-synaptic volume transmission, and some make long range projections to other brain regions (McDonald et al., 2012; Capogna, 2014; McDonald and Zaric, 2015). Below we use the current immunohistochemical classification to review neuronal subsets and known circuit connections in the BLA.

### Parvalbumin GABAergic Neurons

Parvalbumin expressing neurons form ~40–50% of the total interneuron pool and largely target the peri-somatic region of principal neurons (Spampanato et al., 2011). The density of PV+ varicosities is highest in the magnocellular and parvicellular portion of basal nucleus and dorsolateral and ventrolateral regions of the lateral amygdala (McDonald and Betette, 2001). Nearly half these cells also express calbindin (McDonald and Mascagni, 2002). Anatomically, PV+ cells have been divided into three types that target different cellular compartments (McDonald and Mascagni, 2002; Woodruff and Sah, 2007b; Vereczki et al., 2016). The majority (~50%) of PV+ interneurons are basket cells and they characteristically make synaptic contacts on the soma and proximal dendrites of principal neurons (Woodruff and Sah, 2007b; Vereczki et al., 2016). The second group, called axo-axonic or chandelier cells, although less numerous, form a distinct population that make a characteristic string or “cartridge” of synaptic contacts on the axon initial segment of principal neurons, the key site of action potential initiation (Woodruff et al., 2006; Bienvenu et al., 2012; Veres et al., 2014; Spampanato et al., 2016). The third group target principal neurons and also send axon collaterals to amygdala-striatal region transition zone, named as AStria cells (Bienvenu et al., 2012). Finally, some PV+ interneurons also innervate other PV+ neurons, and SST neurons (Muller et al., 2005; Spampanato et al., 2016). Electrophysiologically, PV+ cells are often described as a single population–called fast spikers (FS cells) with narrow action potentials (half-width of ~0.5 ms) which can discharge at high frequencies (200–300 Hz) with little to no spike frequency adaptation (Ascoli et al., 2008). However, in the BLA, in line with the anatomical separation, they have been divided into several subtypes based on their different patterns of firing to long current injection, and gap-junctional coupling (Rainnie et al., 1991; Woodruff and Sah, 2007b). Of these, cells which show little or no spike frequency adaptation (FS cells) have been proposed to be basket cells targeting the somatic region, while cells with spike frequency adaptation target distal dendrites (Woodruff and Sah, 2007b). Finally, chandelier cells that target axon initial segment, predominantly show a fast-stuttering phenotype (unpublished observations). PV+ interneurons receive excitatory input from both principal neurons, and cortical sources thus forming both feedback and feed-forward inhibitory circuits (Samson et al., 2003).

Although GABAergic transmission in mature neurons is generally thought to be inhibitory, PV chandelier cells in the BLA have been reported to have excitatory action on at least some principal neurons driving both feedback and feedforward excitatory connections (Woodruff et al., 2006; Spampanato et al., 2016). Thus, it has been suggested that in the BLA, some principal neurons are driven to threshold by chandelier neurons (Woodruff et al., 2006). However, the identity of principal neurons excited by chandelier cells, the mechanism of this excitatory drive, or if these cells form a distinct population of principal neurons is not known. Each chandelier cell is estimated to innervate several hundred principal neurons, thus individual cells can powerfully influence glutamatergic drive in the local circuits (Bienvenu et al., 2012; Veres et al., 2014; Spampanato et al., 2016). In addition to chemical synapses, PV+ cells are extensively interconnected with similar types connected by gap junctions, thus are ideally positioned to generate synchronized network activity (Woodruff and Sah, 2007b).

### Somatostatin GABAergic Neurons

Somatostatin expressing interneurons predominantly target the distal dendrites and influence the gating of excitatory synaptic input to principal neurons (Muller et al., 2007; Wolff et al., 2014). These cells receive inhibitory input from PV+ basket cells and VIP+ cells (Wolff et al., 2014; Krabbe et al., 2019). A proportion of SST+ neurons, including some that colocalize with neuropeptide Y, send long range axonal projections out of the BLA (McDonald et al., 2012; McDonald and Zaric, 2015). Finally, a small population of SST+ cells have been identified as neurogliaform cells which mediate local slow inhibition by diffuse volume transmission (Manko et al., 2012). Thus, as for PV+ interneurons, SST+ cells show a diversity of different types that likely play distinct functional roles. Electrophysiologically, individual SST+ neurons typically display accommodative action potential discharge. It is interesting that PV+ interneurons that target distal dendrites also have accommodating discharge properties (Woodruff and Sah, 2007b), and these two cells types are electrophysiologically indistinguishable. The computational relevance of distal dendrite targeting interneurons having an accomodating phenotype is not currently clear.

### Cholecystokinin/Vasoactive-Intestinal Peptide GABAergic Neurons

Cholecystokinin expressing interneurons have been classified into two subsets: Large (CCK_L_) and small (CCK_S_) neurons (Mascagni and McDonald, 2003). CCK_L_ cells co-express calbindin or VGLUT3 and are the only cell type to express cannabinoid receptor 1 (CB1) in the BLA (Katona et al., 2001; Rovira-Esteban et al., 2017), while CCK_S_ cells co-express calretinin or VIP. Electro-physiologically, CCK cells reported to display three distinct spike discharge patterns and broader action potentials than principal neurons (Rovira-Esteban et al., 2017). Both subtypes form peri-somatic synaptic contacts on the post-synaptic principal neuron and have been reported to drive both fast GABA_A_ receptor mediated and slow GABA_B_ mediated inhibitory responses that provide peri-somatic inhibition to principal neurons and form feedback inhibitory circuits (Vereczki et al., 2016; Veres et al., 2017; Krabbe et al., 2019).

VIP+ neurons form a heterogeneous group of cells that are sparsely distributed throughout BLA (Rhomberg et al., 2018) and target principal neurons and other interneurons. Nearly 60% of VIP+ cells co-express calretinin and these cells target PV+, SST+, CCK+, as well as neurogliaform and other VIP interneurons (Rhomberg et al., 2018). Some VIP+ neurons co-express CCK/CB1 and innervate the soma of principal neurons. The remaining population of VIP+ cells do not appear to express any other known markers and target interneurons. Thus, VIP+ interneurons are a complex set of cells that mediate both feedforward and feedback inhibition, and as a result of their strong connections to other interneurons also form local disinhibitory circuits.

## Functional role of GABAergic Cell Types in Fear Memory

A large body research over many years has established that cellular activity and synaptic plasticity in the BLA underpin emotional learning and memory, particularly of aversive events (LeDoux, 2003). The neural circuits that underpin emotional memory formation and retrieval has been extensively investigated using cued Pavlovian fear conditioning. In this paradigm, a neutral conditioned stimulus (CS, such as auditory tone) is paired with an aversive unconditioned stimulus (US, typically a mild foot shock), such that after several pairings, the CS elicits a defensive “fear” response with motor, autonomic and neuroendocrine components. However, subsequent repeated unpaired presentation of CS alone leads to reduced conditioned response, known as “fear extinction.” Fear extinction is thought to result from the formation of new associative memory representation (Maren and Quirk, 2004; Quirk and Mueller, 2008; Marek et al., 2019). Fear acquisition, storage and extinction engage the amygdala through evolutionarily conserved circuits and provides a robust behavioral paradigm to investigate neurobiological correlates of learning and memory (Fanselow and Poulos, 2005; Fanselow and Wassum, 2015). These studies have established that excitatory inputs from the thalamus and the cortex carrying sensory information encoding the CS converge on principal neurons in the dorsal part of the BLA, the lateral amygdala (Polepalli et al., 2020; Sun et al., 2020). Inputs carrying CS and US information converge on neurons in the BLA (Romanski et al., 1993; Wolff et al., 2014; Windels et al., 2016). The BLA also receives multimodal information from the ventral hippocampus and prefrontal cortex (McDonald, 1998; Sah et al., 2003). Together with the CS and US, this information is processed by local circuits in the BLA and the resultant plasticity of excitatory inputs carrying CS information is thought to underlie this associative learning (Pape and Pare, 2010; Sun et al., 2020). Subsequent presentation of the CS again recruits circuits within the BLA that sends afferents to the central amygdala, from which downstream projections drive the defensive response - fear expression (Tovote et al., 2015). Within the BLA, sensory and multimodal inputs drive potent feedforward inhibition to principal neurons (Lang and Pare, 1997; Windels et al., 2010; Krabbe et al., 2018). Thus, it has been recognized that local GABAergic circuits gate principal neuronal activity during both acquisition and recall of fear memory (Tovote et al., 2015).

Early experiments established the important role of local inhibition in fear learning (Harris and Westbrook, 1998; Ehrlich et al., 2009), and cortical inputs to interneurons in the BLA also show synaptic plasticity (Mahanty and Sah, 1998; Spampanato et al., 2011). The development of transgenic lines to identify selective neural populations (Huang et al., 2014), and molecular tools to temporally control neural activity (Johansen et al., 2012) is now providing rich insight into the functional roles of specific interneuron populations. Thus, Wolff et al. (2014) combined optogenetics with single unit recordings targeted at PV+ and SST+ neurons in the BLA during fear conditioning (Wolff et al., 2014). They found that presentation of an auditory CS drives PV+ interneurons but inhibits SST+ interneurons, while the US (footshock) inhibits both PV+ and SST+ interneurons. Behaviourally, activating PV+ cells during associative fear learning (i.e., CS-US pairing) reduced learning to the CS+, showing that the activity of PV+ interneurons is necessary for effective learning. In support of this, artificially activating PV+ cells were during CS presentation, enhanced learning while inhibition of these cells during the CS reduced fear memory. In contrast, manipulation of SST+ interneurons had the opposite effect. Driving these cells during CS+ presentation reduced learning and inhibiting SST+ cells had the opposite effect. These results show that both PV+ and SST+ interneurons are engaged during associative fear learning. Authors proposed that that during CS-US pairing, there is effective disinhibition of principal neurons firstly by PV+ cells inhibiting SST+ cells during the CS and then by inhibition of both interneuron populations during the US (Wolff et al., 2014).

Targeting a different interneuron population, Krabbe et al. (2019) found that VIP+ cells also modulate fear learning. Targeted expression of the genetically encoded calcium sensor GCaMP to detect spike triggered calcium transients, revealed that either the CS or the US can recruit VIP+ interneurons in the BLA, but the US alone or combined CS+US presentation recruited a substantially larger proportion of VIP+ cells, as compared to CS alone. Unexpectedly, in the BLA, optical activation of VIP+ interneurons enhanced putative principal neuronal discharge. Behaviourally, inhibition of VIP cells during the US reduced learning with a lower freezing response on fear retrieval, showing that activity of VIP+ cells during learning is needed for optimal learning. Thus, they concluded that VIP cells drive disinhibitory circuit operations in the BLA in the fear conditioning paradigm. Thus, optogenetic targeting of GABAergic subtypes defined by cytosolic proteins revealed putatively distinct functional role for PV+, SST+, and VIP+ neurons in the Pavlovian fear conditioning paradigm. Taken together, these studies propose both PV+ and VIP+ neurons operate through disinhibitory circuit mechanisms during associative learning of CS and US. Based on their synaptic connectivity in the BLA, it is proposed that PV+ gate CS by inhibiting SST+ cells (Wolff et al., 2014) and VIP+ cells mediate disinhibition mainly during US (Krabbe et al., 2019).

These experiments show that PV+, SST+, and VIP+ interneurons are activated differently by the auditory CS and footshock, and have different effects on fear learning. However, as described above, both PV+ and VIP+ cells can be subdivided into several families with distinct circuit functions. To date, SST+ interneurons are less well-studied but may also contain several types. These different types are likely to have different functional roles and raise many questions. For example, PV+ cells have been separated into basket cells, axo-axonic chandelier cells and AStria cells. Juxta-cellular, *in vivo* recordings in anesthetized animals have found that aversive stimuli (hind paw pinch or foot shock) evoke a heterogeneous response in PV basket cells, depress PV+ AStria cells, whilst robustly increase discharge of PV+ chandelier cells (Bienvenu et al., 2012). It is noteworthy that some PV+ chandelier cells have been reported to recruit principal neurons and drive feedback and feedforward circuits *in vitro* (Woodruff and Sah, 2007b; Spampanato et al., 2016). As chandelier cells are preferentially recruited by the US (Bienvenu et al., 2012), whether they operate *in vivo* to recruit PNs for encoding fear memory is not known. Moreover, basket type PV+ cells form the main subtype of GABAergic neurons that provide strong somatic inhibition on principal neurons and tightly control spike discharge (Woodruff and Sah, 2007b; Vereczki et al., 2016). The CS mediated recruitment of PV+ cells is proposed to disinhibit principal neuronal dendrites by inhibition of SST+ cells (Wolff et al., 2014). However, it remains unclear how PV+ basket cell mediated disinhibition of dendrites overcomes direct peri-somatic inhibition of principal neurons by the same cell types to facilitate recruitment and plasticity.

During fear learning, BLA circuits generate oscillatory network activities that entrain local principal neurons (Pare et al., 2002; Harris and Gordon, 2015), which provide output to the hippocampus and prefrontal cortex and generate coherent activities to encode emotional memory (Likhtik et al., 2014). Microcircuit mechanisms for the generation of network oscillations have been extensively studied in the hippocampus. In the hippocampus, feedback and feedforward GABAergic circuit motifs formed PV subset proposed to orchestrate network oscillations (Freund and Buzsaki, 1996; Buzsaki, 2015; Roux and Buzsaki, 2015). While network oscillations in the amygdala are clearly important in fear learning (Pare et al., 2002; Sah et al., 2003), the role of different interneurons in coordinating these different oscillatory bands in the BLA is not currently well-understood. *In vivo*, PNs in the BLA fire rarely even in the presence of extrinsic stimuli and are tightly controlled by local inhibitory circuits (Windels et al., 2016). Incoming inputs to the BLA have been reported to drive feedforward GABAergic neurons during oscillatory network activities and control the spike-timing of PNs (Bazelot et al., 2015). However, how different GABAergic neuronal subtypes and their intricate circuit motifs operate during network oscillations in the BLA remain elusive. Targeted optical manipulation using cytosolic marker proteins in GABAergic subtypes in the BLA has revealed a broad picture of function role of GABAergic cell-types in fear memory. Future studies need to identify functional roles for these GABAergic cell-types and their circuit motifs in the generation of oscillatory network activities in the BLA.

## Conclusion and Future Directions

The BLA forms a major part of the amygdaloid complex, a region that has a key role in emotional regulation, learning and memory. Dysfunctions within BLA circuits are thought to contribute to a host of anxiety-like disorders such generalized anxiety and post-traumatic stress (Perumal et al., 2018). GABAergic circuits in the BLA play a central role in all aspects of its function and compounds such benzodiazepines that are first line anxiolytics modulate inhibitory synaptic transmission. Thus, understanding GABAergic circuits in the BLA will not only provide insight into learning and memory formation, but also reveal pathological mechanisms associated with dysfunction. The development of genetically driven markers and opto/chemo genetic tools to control and manipulate targeted population of neurons is now rapidly advancing our understanding of processing within the BLA (Janak and Tye, 2015; Mobbs et al., 2019). It is becoming increasingly clear that interneurons are far more diverse and complex than initially thought and our understanding of the roles of GABAergic neuronal subtypes associated with different network functions in the BLA is just scratching the surface. This understanding is pivotal to identify how different circuit motifs operate during physiological network activity and unravel computational principles for emotional memory. A clearer identification of cell types in the BLA, their connections, and the receptors present in different cell-type and sub-cellular compartments will profoundly influence our understanding of the circuits that underpin learning and memory and mediate the different behavioral states in emotional regulation (Perumal et al., 2018).

## Author Contributions

All authors listed have made a substantial, direct and intellectual contribution to the work, and approved it for publication.

## Conflict of Interest

The authors declare that the research was conducted in the absence of any commercial or financial relationships that could be construed as a potential conflict of interest.
